# Global health governance performance during Covid-19, what needs to be changed? a delphi survey study

**DOI:** 10.1186/s12992-023-00921-0

**Published:** 2023-03-31

**Authors:** Wafa Abu El Kheir-Mataria, Hassan El-Fawal, Sungsoo Chun

**Affiliations:** Institute of Global Health and Human Ecology, The American University in Cairo, P.O. Box 74, New Cairo, 11835 Egypt

**Keywords:** Global Health, Governance, Equity, Covid-19, Delphi, Performance

## Abstract

**Background:**

Covid-19 is not the first pandemic to challenge GHG. Preceding outbreaks and epidemics were sources of continuous debate on GHG leadership and structure resulting in its current structure. However, Covid-19 proved the presence of many deficits in the current GHG. The response to the Covid-19 pandemic is a cumulative result of all policies and actions of different governments and agencies active in global health. Assessing how Covid-19 is being handled globally provides lessons for ensuring better performance in facing upcoming outbreaks. This study has three main objectives: first, to evaluate the performance of GHG during Covid-19 in general and in relation to Covid-19 vaccine equity in particular. Second, to identify the reasons behind this performance; and third, to propose prospective changes in GHG for better performance.

**Methods:**

A cross-sectional research design using the Delphi method was applied. A panel of experts participated in the three-round Delphi surveys. Their scores were used to perform consensus, performance and correlation analysis.

**Results:**

GHG performance limited the achievement of Covid-19 vaccines’ global equity. GHG performance is a product of the existing GHG system, its actors and legal framework. It is a collective result of individual GHG actors’ performance. The most influential actors in decision-making regarding Covid-19 vaccine are the vaccine manufacturers and governments. While the most invoked power to influence decision are economic and political powers. Covid-19 decisions underlying value, although had human right to health at the base, overlooked the concept of health as a global public good and was skewed towards market-oriented values. GHG mal-performance along with its underlying factors calls for four main changes in GHG structure: assigning a clear steward for GHG, enhanced accountability, centralized authority, more equitable representation of actors, and better legal framework.

**Conclusion:**

GHG structure, actors’ representation, accountability system, and underlying priorities and value require future modification for GHG to achieve better future performance and higher health equity levels.

## Introduction

Right from the start of the Covid-19 pandemic, scientists recognized the zoonotic nature of the disease, as caused by a virus transmitted from animals to humans [1], and the risk of its global spread. Equally, many suspected that with the climate and environmental changes that the world is living through, humans would continue to encounter other zoonotic infectious diseases [2] threatening their lives and livelihoods. Assessing how Covid-19 is being handled globally provides lessons for ensuring better performance in facing upcoming outbreaks. Deficits such as poor coordination and regulatory overlapping [3], a fragile system of global health governance [4], and vaccine inequity[5] in the current GHG system resurfaced during the current Covid 19. These deficits intrigued researchers and policymakers to search for underlying reasons and propose changes for better outcomes in the future.

The response to the Covid-19 pandemic is a cumulative result of all policies and actions of different governments and agencies active in global health. These actors fall, by various scholars’ definitions, under the umbrella of the Global Health Governance (GHG) system [6–9]. Thus, the performance in the Covid-19 response ought to be assessed at a global level including the actors and the factors affecting their performance, such as their interests and capacities, as well as the various components of the GHG system, such as the legal framework.

Covid-19 is not the first pandemic to challenge GHG. Preceding outbreaks and epidemics were sources of continuous debate on GHG leadership and structure. They made politicians recognize the global health danger they face, shifting global health from low politics to high politics [10]. This resulted in the current structures and finance system of GHG the way we know it today. Nevertheless, COVID-19 is placing tremendous pressure on GHG leaders like never before. It provided a test of the effectiveness of the current GHG in performing its role. From ordinary people to heads of States, all are questioning the structure, functions, power, and ability of the existing GHG to ensure global health security by protecting populations’ health.

Certain speculations are made regarding upcoming changes following Covid-19 [11]. Many of the GHG challenges and recommendations raised during the COVID-19 period were raised through reviews and openings [3–5], and there were not many analytical studies by systematic and designed collective opinions by experts. Delphi surveys is a valuable method to collect diverse experts’ opinions and viewpoints on core areas of challenges and prospective modifications in GHG. The main use of the Delphi method is to reach a consensus on debatable issues [12–14]. The Delphi method allows for reaching a consensus on the most important points while avoiding group dynamics where some participants dominate the discussions [15]. The Delphi method can also be used to predict future events or changes. It is used when experts’ opinions are the only source of information [16]. Thus, since the Delphi method was introduced by RAND Corporation in the 1950s [9], it has been used in various disciplines, such as social sciences [10] and health sciences [11–14], especially in case of planning and structuring for expert discussions to generate insights on debatable issues with little information [15].

This study has three main objectives: first, to evaluate the performance of GHG during Covid-19 in general and in relation to Covid-19 vaccine equity in particular. Second, to identify the reasons behind this performance; and third, to propose prospective changes in GHG for better performance.

## Methods

### Research design and questionnaire

This study is a cross-sectional research design using the Delphi method. Data was collected using the three-round Delphi surveys.

The Delphi survey questionnaire was composed of eight main questions, each question has a set of statements. The total number of statements in the study was 72 statements. The questions were based on eight previously identified core areas extracted from a systematic review of the literature produced on global governance and health equity in the context of Covid 19 [17] as well as on a literature review of models and theories on governance in general and global health governance in particular. The eight core areas are: GHG performance in the current Covid-19 pandemic focusing on Covid 19 vaccines, Covid-19 vaccine equity as handled by the GHG, factors affecting countries’ ability to acquire Covid-19 vaccines, GHG current structure as the main factor contributing to Covid-19 vaccine equity, GHG regulatory framework in relation to global justice and equity, GHG actors’ underlying values and priorities in managing Covid-19 vaccines, decision-makers, their interest and the power they use in the GHG arena, and finally characteristics for future changes in GHG. The questionnaire was pilot tested on three experts to assess survey conditions and the questions’ validity.

### Recruitment and participants

Purposeful sampling was used to recruit experts to participate in the study at a primary stage. The rest of the Delphi panel members were selected through a snowballing technique where the primary participants were solicited to recommend other experts’ names to be part of the study. The panel members were selected based on their expertise and experience in the field, considering the proportion of the representatives of international organizations, governments, NGOs, and Academia by continent. Sixty Delphi panel members were invited to participate in the study. An introductory email was sent to a total of 60 experts. The email thoroughly explained the study in hand: its aim, objectives, mode (via email), timeline, ethical considerations, and voting principles (the vote on each statement should be based on either the participants’ own opinion or the organization they represent). The Delphi questionnaires were administered using e-mail. Regular reminder emails were sent to assure survey completion.

According to the recommendations for the Delphi surveys, the target number of participants in the study should be around 18 and the minimum accepted number of participants is 10 [18]. For the current study, the final number of participants was 30. Recruited participants presented four main groups of stakeholders: academia, governments, international non-governmental organizations, and United Nations agencies, with years of experience in the field of GHG that ranges between 7 and 50 years (Table 1).

**Table 1:** Experts panel demographics

	Group	No.		%
Age	30–40	3		10
	41–50	4		13.3
	51–60	12		40
	> 60	8		26.7
Organization type	UN system	5		16.7
	Government	6		20
	International organization	2		6.7
	Non-governmental organization	2		6.7
	Academia	USA	5	16.7
		Europe	3	10
		East Asia	1	3.3
		East Mediterranean	6	20
Years of experience	≤ 10	2		6.7
	11–20	5		16.7
	21–30	10		33.3
	31–40	7		23.3
	> 40	4		13.3

### Delphi consensus process

The Delphi survey was done in three rounds. All statements were included in each round. Participants were to vote on the Delphi survey statement. A 7-point Likert Scale was used, with one as the lowest score possible and seven as the highest available score. Following each round, the average and the standard deviation of participants’ scores on each statement were calculated. The values were then made available to the participants anonymously. They were included in the following round’s questionnaire allowing participants to provide comments and suggestions. In the second and third rounds, the participants were notified to consider the average score and the standard deviation of the previous round before deciding if they were to keep their original scores or they will change them. The difference in the average and standard deviation between the first and second round was minimal while following the second round all participants maintained their scores except for one who changed a few scores. This indicated that experts were confident of their scores and would not change them signaling that the panel members had reached consensus [19]. The response rate in the 2nd round was 96.7% while in the 3rd round it reached 83.3%.

### Data analysis

#### Consensus criteria and calculations

The standard deviation served as a measure of reaching an agreement point or consensus in the current study. According to the literature, the mean and standard deviation can be used as measures of consensus [19, 20]. The breakpoint for agreement using the mean and standard deviation from a 7-point Likert scale is not common, it is more common for a 5-point Likert scale. In a 5-point Likert scale the cutting point is when the mean equals or is greater than 3.25 and the standard deviation is equal to one or less than one [21]. Since this study uses a 7-point Likert scale, the values for the means and standard deviations as breaking points of agreements were recalculated.

The mean for our 7-point Likert scale was calculated as follows [22], giving the value of 4.38:

x_7_ = (x_5_ – 1) (6/4) + 1.

x_7_: Mean for the 7-point Likert scale.

x_5_: Mean for the 5-point Likert scale.

As for the standard deviation, it was calculated using the coefficient of variance concept. Assuming that the coefficient of variance for the given scores on the two scales is equal then the standard deviation for the 7-point Likert scale would be 1.35.

Given the manner in which this survey was conducted – where the participants were asked if they agree with the mean score of the previous round’s score – the mean cannot be used as measure of consensuses. Only the standard deviation can be used as a measure of consensus as it measures the dispersion of the scores from the mean score. If the dispersion is high (SD > 1.35) that means that the participants did not have consensus on this score.

#### Assessing the performance of GHG

Out of the eight questions of the survey, two questions with 16 statements were allocated to assess GHG performance. These statements were scored, and each statement’s mean and standard deviation were calculated. The mean score for each statement was the measure used to assess GHG performance. In this study the value of the mean is not a measure of consensus but rather a measure of agreement with the statement, the higher the score the stronger the agreement, also the higher the mean the better the performance of GHG in that certain point.

#### Future changes in GHG structure and the underlying factors

In the survey one question with five statements was dedicated for depicting areas of future change in GHG structure. These statements were scored and the mean and standard deviation for each statement was calculated to have consensus on the proposed changes and to figure the scores given by the panel which would serve as an indicator of the importance of the proposed change. A correlation analysis was also performed between the proposed areas of change in GHG and the presumed underlying causes of GHG malperformance. The correlation analysis results were used to explain the future changes.

## Results

The Delphi survey included seventy-two statements within which fifty-seven statements gained consensus by the expert panel while the remaining fifteen statements did not gain consensus (Table 2). The absence of consensus on these fifteen statements demonstrates the variation in the panel members affiliation and experience in the field which enriches the results. It also demonstrates that Delphi survey was properly conducted and group dynamics where some participants dominate the discussions was avoided.

**Table 2:** Consensus and correlation values

			8. Characteristics for future changes in GHG				
			8.1 Clear stewardship M = 6.1 SD = 1	8.2 Enhanced accountability M = 6.1 SD = 0.9	8.3 Centralized authority M = 4.6 SD = 1.3	8.4 More equitable representation of actors M = 6.2 SD = 1	8.5 Better legal framework to ensure accountability, information and technology sharing. M = 5.9 SD = 1.1
**1. GHG performance in the current Covid-19 pandemic focusing on Covid 19 vaccines**	M=	SD=					
Generate a collective response to meet the need for the Covid-19 vaccine	3.9	1.2	− 0.344	− 0.421*	− 0.163	− 0.569**	− 0.359
Manage Covid-19 vaccine production	3.8	1.3	− 0.123	− 0.294	− 0.169	− 0.491	− 0.052
Manage Covid-19 vaccine procurement	3.7	1.0	− 0.523**	− 0.433*	− 0.260	− 0.587**	− 0.371
Manage Covid-19 vaccine distribution	3.4	1.2	− 0.364	− 0.279	− 0.123	− 0.559**	− 0.397*
Produce inclusive decisions and guidelines for Covid-19 vaccines	4.9	1.0	0.028	− 0.161	− 0.083	− 0.434*	− 0.063
Produce clear policies and guidelines for countries	4.6	1.1	0.011	0.030	− 0.201	− 0.029	0.073
Produce feasible policies and guidelines for every nation	3.8	1.2	− 0.202	− 0.110	− 0.110	− 0.221	− 0.118
Facilitate global solidarity through managing Covid-19 vaccine (production, procurement and distribution)	3.3	1.2	− 0.053	− 0.245	− 0.149	− 0.451*	− 0.003
GHG overall performance	3.6	0.9	− 0.180	− 0.314	− 0.143	− 0.589**	− 0.164
**2. GHG performance in Covid-19 Vaccine Equity**							
Covid-19 vaccine production (manufacturing) ensured equity across nations in securing the vaccine for their populations	2.5	1.3	− 0.352	− 0.414*	− 0.097	− 0.605**	− 0.341
There is an equal opportunity for every nation to procure the needed amount of Covid-19 vaccines to cover its population	2.2	1.3	− 0.368	− 0.460*	− 0.051	− 0.663**	− 0.327
The Covid-19 vaccine is equitably distributed among nations	2.1	0.9	− 0.483*	− 0.354	− 0.132	− 0.583**	− 0.422*
Using digital and medical technology can enhance Covid-19 vaccine equity	4.4	1.2	0.139	0.059	0.064	− 0.282	0.048
COVAX initiative enhances Covid-19 vaccine equity	4.4	1.2	− 0.018	− 0.126	− 0.213	− 0.307	− 0.093
Actors bared in mind the collective benefit of their actions	3.0	0.9	0.039	0.088	0.254	− 0.306	− 0.033
Actors showed solidarity actions in their decisions regarding the Covid-19 vaccine	3.0	1.0	− 0.036	0.007	0.077	− 0.360	− 0.227
**3. Factors affecting countries’ ability to acquire Covid-19 vaccines**							
Having the knowledge and technology to develop or produce the vaccine	5.0	1.1	0.180	0.327	− 0.148	0.065	0.144
Level of economic and political power a country holds	6.0	0.8	− 0.201	− 0.113	0.075	0.255	0.046
The country’s health system’s capacity to handle the Covid-19 vaccine	5.1	1.1	− 0.225	0.138	0.276	0.013	− 0.112
Bilateral deals to acquire Covid-19 vaccine	4.9	1.2	− 0.060	0.045	0.347	− 0.057	− 0.156
The COVAX initiative	4.1	1.1	0.093	− 0.101	0.200	− 0.313	0.017
Pharmaceutical companies’ interest in financial gain	6.2	1.1	0.156	0.088	0.117	0.611**	0.124
Laws on intellectual property rights	4.5	1.5	− 0.267	0.019	0.164	− 0.109	− 0.141
Country’s representation and influence in GHG	4.8	1.4	− 0.100	0.020	0.295	0.018	0.200
**4. GHG structure and the achievement of Covid-19 equity**							
It is not clear which GHG actor holds the stewardship position (setting priorities, building consensus, setting rules, and evaluating members)	4.6	1.1	0.078	− 0.055	0.029	0.037	− 0.053
The GHG structure is loose with no specified roles and accountability measures	5.1	1.2	0.334	0.318	− 0.094	0.596**	0.385*
Authority is better to be centralized in GHG to ensure the better authority	4.3	1.3	0.093	− 0.175	0.776**	− 0.245	− 0.197
Better representation of countries from the global south in GHG to ensure equity	6.2	0.9	0.148	0.206	− 0.025	0.524**	0.186
Develop a mechanism to monitor the influence of private actors and non-governmental financing organizations in policymaking	6.0	0.9	0.313	0.354	− 0.113	0.542**	0.276
The World Health Organization should have more authority	4.9	1.5	0.122	− 0.068	0.075	− 0.165	− 0.018
WHO should focus on its technical role of providing guidelines	4.8	1.5	− 0.173	− 0.222	0.197	0.022	− 0.291
The role of the World Health Organization should change	5.4	1.2	0.116	0.150	− 0.039	0.089	0.196
United Nations headquarter should hold the stewardship position in GHG	3.6	1.5	− 0.058	− 0.142	0.483*	− 0.012	0.026
Global NGOs should have authority in GHG	3.9	1.5	− 0.127	0.014	− 0.052	0.262	0.081
**5. Laws and regulations of GHG**							
The legal instruments in GHG assure legal accountability of actors	3.3	1.1	− 0.398*	− 0.535**	− 0.208	− 0.447*	− 0.355
The legal instruments in GHG ensure health equity	3.1	1.2	− 0.303	− 0.431*	− 0.202	− 0.429*	− 0.337
International Health Regulations (IHRs) need to be updated	6.1	0.8	0.354	0.439*	− 0.089	0.199	0.484*
IHRs need better enforcement	6.4	0.7	0.446*	0.367	0.153	0.054	0.264
More laws and regulations are needed to regulate actors, their contributions and their interaction	5.5	1.2	0.066	0.048	0.396*	0.168	0.191
**6. Underlying values and priorities in managing Covid-19 vaccines**							
Human rights and the right to health are the main values considered by GHG actors concerning the Covid-19 vaccine	3.9	1.2	− 0.089	− 0.016	0.006	− 0.150	− 0.242
Market-oriented health norms are affecting GHG decisions and actions concerning Covid-19 vaccines	5.6	1.0	0.201	0.021	− 0.159	0.233	0.162
Health as a common good. This concept is being considered in decisions concerning Covid-19 vaccine distribution	3.7	1.0	− 0.196	− 0.122	− 0.101	− 0.563**	− 0.392*
The vulnerability of countries is considered in Covid-19 vaccine distribution to limit the spread of the disease.	2.8	1.0	− 0.124	− 0.085	− 0.089	− 0.457*	− 0.214
**7.1 Who makes / influences decisions regarding the Covid-19 vaccine?**							
WHO - World Health organization	4.2	1.1	0.199	0.327	0.399*	− 0.056	0.187
UNICEF - United Nations International Children’s Emergency Fund	3.2	1.2	− 0.012	0.221	0.113	0.016	0.111
GAVI - Global Alliance for Vaccines and Immunization	4.2	1.4	0.120	0.285	0.199	0.137	0.200
CEPI - Coalition for Epidemic Preparedness Innovations	3.7	1.1	− 0.031	0.473*	− 0.085	0.413	0.262
Bill & Melinda Gates Foundation	4.4	1.1	0.034	0.170	− 0.138	0.074	0.019
The World Bank	4.2	1.3	0.263	0.360	0.274	0.028	0.315
Research agencies	3.4	1.1	0.134	0.175	− 0.167	− 0.064	0.204
Vaccine manufacturers	5.7	1.2	0.217	0.502**	0.092	0.618**	0.417*
Governments	5.5	1.1	− 0.081	− 0.149	− 0.111	− 0.260	− 0.246
Non-governmental Organizations	2.8	0.7	− 0.094	− 0.089	0.055	− 0.127	− 0.130
**7.2 What forms of power do they invoke?**							
Political influence	5.7	1.1	0.478*	0.221	0.082	0.136	0.494**
Economic power (market and trade relations, material capital)	6.1	0.8	0.033	0.031	− 0.134	0.372	0.291
Technical expertise (Knowledge and technology)	4.9	0.8	− 0.132	− 0.139	− 0.079	− 0.116	− 0.081
Cultural capital	3.0	1.2	− 0.302	0.125	− 0.498*	− 0.055	− 0.102
**7.3Whose interests are at stake?**							
WHO - World Health organization	5.5	1.3	0.512**	0.513**	− 0.019	0.334	0.467*
UNICEF - United Nations International Children’s Emergency Fund	4.6	1.8	0.340	0.617**	0.084	0.435*	0.401
GAVI - Global Alliance for Vaccines and Immunization	4.5	1.8	0.435*	0.602**	0.044	0.120	0.464*
CEPI - Coalition for Epidemic Preparedness Innovations	3.8	1.6	0.187	0.457*	− 0.015	0.224	0.285
Bill & Melinda Gates Foundation	4.2	1.5	0.232	0.359	0.508**	0.032	0.213
The World Bank	4.0	1.5	0.130	0.299	0.437*	0.059	0.167
Research agencies	4.4	1.5	0.396*	0.577**	− 0.092	0.348	0.494*
Vaccine manufacturers	5.2	1.9	− 0.051	0.226	0.214	− 0.025	0.125
Governments	5.7	1.3	0.129	0.291	0.170	− 0.046	− 0.072
Non-governmental Organizations	3.7	1.5	0.332	0.394	0.461*	0.236	0.276

**correlation is significant at 0.01 level, * correlation is significant at 0.05 level

### GHG performance

**Fig. 1:**
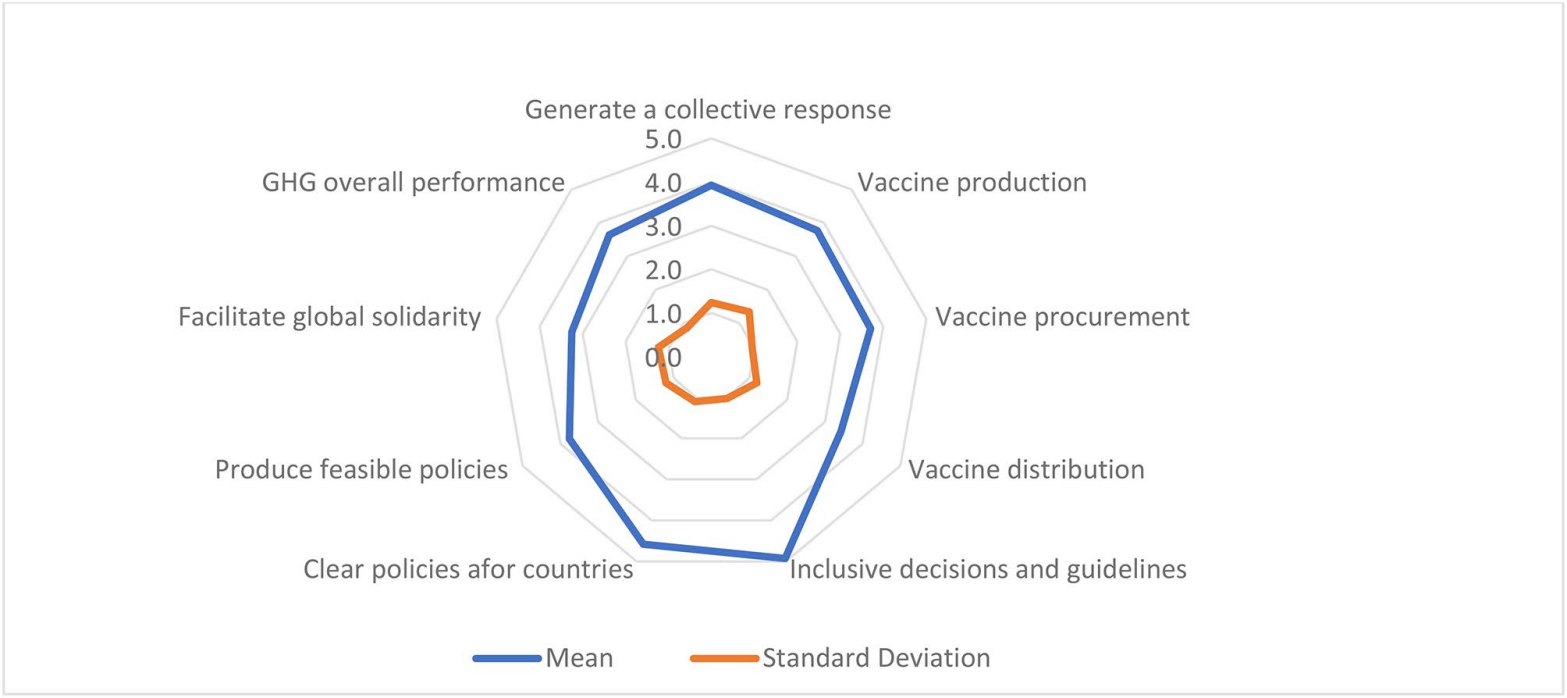
GHG performance in the current Covid-19 pandemic focusing on Covid 19 vaccines

The panel had consensus on all the scores given for the performance of GHG in managing the Covid-19 vaccines during the pandemic as well as on the GHG performance in achieving equity concerning Covid-19 vaccine (Table 2). GHG performance during the Covid-19 in general and in relation to Covid-19 vaccine was assessed using nine statements. The participants’ scores indicated that GHG performance was “disappointing”, the mean scores for the ten statements did not reach five at the 7-point Likert. GHG overall performance mean score (M) was 3.6. Within this generally deficient performance, GHG best performance was in producing *inclusive* decisions and guidelines for Covid-19 vaccines (M = 4.9) followed by the production of *clear* guidelines to countries (M = 4.6), while the worst performance was in facilitating global solidarity (M = 3.3) and in managing vaccine distribution (M = 3.4). GHG ability to manage Covid-19 vaccine production and procurement as well as its ability to generate a collective response and feasible policies was average compared to its performance in other aspects (Fig. 1).

**Fig. 2:**
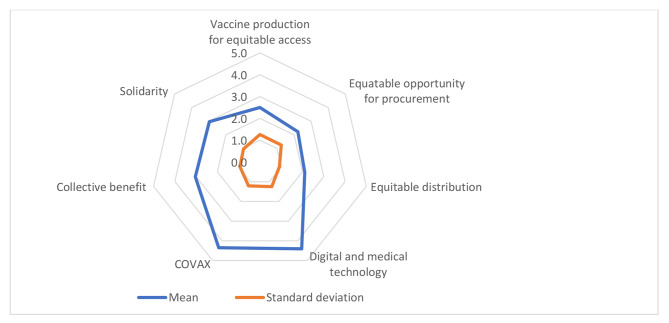
GHG performance in Covid-19 Vaccine Equity

As for GHG performance in achieving equity regarding Covid-19 vaccines and how it can be enhanced, it was similarly “inadequate”. Panel experts decided that Covid-19 production, distribution and procurement were highly inequitable among countries (M = 2.5, 2.1, 2.2 consecutively). They also scored low on the two statements related to GHG actors’ considerations of their actions. Actors poorly considered their solidarity actions (M = 3.0) as well as the collective consequences of their actions (M = 3.0). On the other hand, the panel experts gave slightly higher scores for the COVAX initiative and digital and medical technology as a tool to achieve equity (M = 4.4 for both statements) (Fig. 2).

### Factors affecting countries’ ability to acquire Covid-19 vaccines

Regarding the factors affecting countries’ ability to acquire Covid-19 vaccines, the panel had a consensus on six out of eight statements. The panel had a consensus that the two most important factor that enable countries to acquire the vaccine are pharmaceutical companies’ interest in financial gain (M = 6.2) and the level of economic and political power a country holds (M = 6). The other important factors that the panel had consensus on were the country’s health system’s capacity to handle the Covid-19 vaccine (M = 5.1), having the knowledge and technology to develop or produce the vaccine (M = 5), the bilateral deals to acquire Covid-19 vaccine (M = 4.9), and the COVAX initiative (M = 4.1). The panel did not have a consensus on the scoring of two factors affecting countries to acquire the vaccine, these factors are: the laws on intellectual property rights and the country’s representation and influence in GHG.

### GHG structure and the achievement of Covid-19 equity

On the role of GHG structure in the achievement of Covid-19 equity, the panel had a consensus on six out of ten statements. The panel had a consensus that for GHG structure to support equity has to (from the highest to the lowest important): have a better representation of countries from the global south (M = 6.2), develop a mechanism to monitor the influence of private actors and non-governmental financing organizations in policymaking (M = 6), change the role of the World Health Organization (M = 5.4), have a more controlled GHG structure with specified roles and accountability measures (M = 5.1), clarify which GHG actor holds the stewardship position (M = 4.6), and centralize authority in GHG (M = 4.3). The panel did not have a consensus on the scoring of the following statement: The World Health Organization should have more authority, WHO should focus on its technical role of providing guidelines, United Nations headquarter should hold the stewardship position in GHG, and Global NGOs should have authority in GHG.

### Laws and regulations of GHG

The legal framework of the GHG statements harnessed a full consensus of the panel. They agreed that (from highest to lowest score): the International Health Regulations (IHRs) need better enforcement (M = 6.4), IHRs need to be updated (M = 6.1), more laws and regulations are needed to regulate actors’ contributions and interactions (M = 5.5), the legal instruments in GHG assure legal accountability of actors (M = 3.1), and that the legal instruments in GHG ensure health equity (M = 3.1).

### Underlying values and priorities in managing Covid-19 vaccines

The panel had full consensus on the scoring of the underlying values and priorities in managing Covid-19 vaccines. They scored the following values and priorities used in GHG in decreasing order: market-oriented health norms (M = 5.6), human rights and the right to health (M = 3.9), health as a common good (M = 3.7), and countries’ vulnerability (M = 2.8).

### Who makes/influences decisions regarding the Covid-19 vaccine?

In the area of who influences decisions regarding the Covid-19 vaccine, the panel had a consensus on scores given to all the proposed actors except GAVI (Global Alliance for Vaccines and Immunization). The scores given to the actors influence were in the following order: vaccine manufacturers (M = 5.7), governments (M = 5.5), Bill & Melinda Gates Foundation (M = 4.4), WHO - World Health Organization (M = 4.2), The World Bank (M = 4.2), CEPI - Coalition for Epidemic Preparedness Innovations (M = 3.7), research agencies (M = 3.4), UNICEF - United Nations International Children’s Emergency Fund (M = 3.2), and finally Non-governmental Organizations (M = 2.8).

### Forms of power invoked and whose interest at stake

The panel had also a consensus on the type of power used by these actors. They gave the highest score for economic power (M = 6.1), then political power (M = 5.7), followed by technical expertise (M = 4.9) and lastly cultural capital (M = 3). Coming to whose actor’s interests are at stake, the panel had only consensus for the WHO (M = 5.5) and Governments (M = 5.7).

### Characteristics for future changes in GHG

The final set of findings focuses on the characteristics of future changes in GHG and how they relate to other survey-listed factors that affect GHG performance. The panel had a consensus on all the GHG structural change statements, the highest score was for changing the GHG structure to have a more equitable actors’ representation (M = 6.2), then to clear stewardship (M = 6.1) and enhanced accountability (M = 6.1), followed by having a better legal framework to ensure accountability, information and technology sharing (M = 5.9), and finally centralized authority (M = 4.6).

The five prospective GHG changes were found to correlate with different factors. For GHG to have clearer stewardship was negatively correlated with: GHG performance in Covid-19 vaccine procurement, GHG performance in equitably distributing Covid-19 vaccines, and GHG legal instruments’ ability to hold GHG actors accountable. And positively correlated with: the need for better enforcement of IHRs, the political power used by actors in GHG, and the WHO interest in influencing decision-making in GHG.

The need for enhanced accountability in future GHG structure was negatively correlated with: GHG ability to generate a collective response, GHG performance in managing Covid-19 vaccine procurement, GHG performance in ensuring equity through vaccine production and providing an environment where every nation can procure the needed number of Covid-19 vaccines, the ability of GHG legal framework to hold actors accountable and to ensure equity. On the other hand, it was positively correlated with: the fact that IHRs need to be updated, that CEPI and Vaccine manufacturers are decision-makers in GHG, and that WHO interests in policymaking are at stake.

For future centralization of GHG’s authority, this aspect was negatively correlated with: the use of cultural capital as a form of power in GHG. And positively correlated with: achieving better equity due to GHG centralized authority, having the United Nations headquarter to hold the stewardship position in GHG, the need for more laws to regulate actors and their contributions and interactions, and the fact that WHO is a decision maker in GHG.

For GHG to have a more equitable actors’ representation, this perspective was negatively correlated with: GHG overall performance, GHG performance in generating a collective response to meet the need for the Covid-19 vaccine, managing Covid-19 vaccine procurement and distribution, producing inclusive decisions and guidelines for Covid-19 vaccines, and facilitating global solidarity through managing Covid-19 vaccine. It is also negatively correlated with GHG performance in achieving equity through vaccine manufacturing, procurement and distribution. The GHG legal instruments’ ability to assure legal accountability of actors and equity, the value of health as a common good and the vulnerability of countries as a priority in GHG decisions were also negatively correlated. On the other hand, pharmaceutical companies’ interest in financial gain, the looseness of GHG structure, the need for better representation of countries and for developing a mechanism to monitor the influence of private actors and non-governmental financing organizations in policymaking, the role of vaccine manufacturers in decision-making were all positively correlated.

Lastly, future GHG structure with a better framework was negatively correlated with: GHG performance in managing Covid-19 vaccine distribution in general and in an equitable manner, and health as a common good for GHG actors. And positively correlated with: the GHG current structure being loose, the need for better enforcement of IHRs, the role of vaccine manufacturers in decision-making, the use of political power in GHG decision-making, and finally, WHO interests in decision-making.

## Discussion

Covid-19 pandemic was described as a catastrophe hitting humanity [23–26]. It was large in scale that it brought all actors in the global health arena into action. Actors by nature had different domains of action and different functions in the global health field. In face of the pandemic, each actor hasted to act to its best ability to face the ramifications of the pandemic. All these actors are considered part of the GHG system present today for that GHG is defined as “the use of formal and informal institutions, rules, and processes by states, intergovernmental organizations, and nonstate actors to deal with challenges to health that require cross-border collective action to address effectively” [8]. Governance, on the other hand is described in the literature as the process of exercising authority with the aim of guidance and regulation of the governed so as to achieve common interests. Authority in governance is founded on collaboration, negotiation, and partnership amongst many players thus distrusting the power between actors [27].

Thereafter, the authority as well as the responsibility is distributed among actors based on the concept of partnership and collaboration. Consequently, the performance of GHG is the accumulative results of all actors’ decisions and actions that are influenced by their interests, priorities, values, and power. These actors are not present in the void, they are present in the GHG system. The structure, dynamic and regulatory framework of this system affect the actors conduct. Assessing the performance of GHG entails assessing all the above: the actors’ actions, the influencing factors, as well as, the structure of the GHG system and its regulatory framework. In the current study, GHG governance performance in managing Covid-19 vaccines and in achieving equity in this area is assessed using experts in the field of global health. The expert panel were engaged in three rounds Delphi survey to reach a consensus on these areas of assessment.

The panel had a consensus that GHG had performed poorly. GHG decisions and actions toward handling the vaccines whether it was in their production, distribution and procurement or in the guidelines and policies, did not manage to satisfy many nations’ needs of the vaccine or reach out for an adequate level of solidarity to help these nations. Having this weak performance has led to apparent worldwide inequities regarding the Covid-19 vaccines. However, the Covid-19 vaccines inequity is directly related to the current structure of GHG. According to the panelists, the inequity is due to the loose structure of GHG and the absence or an unclear stewardship. For improved equity, the role of WHO needs to change, authority needs to be more centralized and monitoring mechanisms to hold actors accountable are in need. Moreover, the global south ought to be better presented in the GHG system.

Actors in the GHG can be roughly organized into five groups: the UN agencies with the WHO as the main one, governments, non-governmental organizations, vaccine manufacturers, and the international organizations which can be further divided into funding agencies and research and service agencies. The panel had a consensus that, of these players, the government and vaccine producers have the greatest influence over choices with Covid-19 vaccinations followed by funding agencies.

Vaccine manufacturers are pharmaceutical companies. Pharmaceutical companies are for-profit, market-driven businesses that place little value on the concept that health should be regarded as a common good. Thereafter when economically well-off countries proposed deals to reserve a large number of doses of upcoming vaccines, pharmaceutical businesses concurred and struck agreements with these countries.

Countries’ governments are important actors in GHG, their performance in acquiring and handling Covid-19 vaccine and the policies and measures they adopted contributed to the final GHG performance. Countries’ performance is tied to a number of factors. The panel had consensus that countries’ economic, political and technical power as well as a country’s representation in GHG are determinants in acquiring the vaccines. Technological powers enabled some countries to manufacture the vaccines while economic and political power allowed countries to procure the vaccine and influence decisions regarding the vaccines distribution and affect other countries’ ability to acquire the vaccine. Certain countries used their power to strike bilateral deals to secure their needs of the vaccine regardless of the consequences of these deals on other countries’ ability to acquire the vaccine [28]. Also, capacity of the countries’ health systems in terms of facilities, human and financial capacity are detrimental for procuring, storing and administering the vaccines. Certain African countries did not have the facilities nor the capacities to store and administer the vaccines resulting in low vaccine accessibility [29].

Funding agencies namely GAVI, Bill and Melinda gates and the World Bank are major actors in GHG and according to the panel have moderate influence on decisions regarding the vaccines. The financial support that they can provide is the source of their power. Gavi is a key partner in the COVAX initiative which is considered an enabler to acquire the vaccine. With the funds Gavi provides, many poor countries were able to acquire the vaccine despite their weak economic, technological and political powers.

According to the panel scoring, WHO which is recognized as one of the most important GHG actor did not have the upper hand in decision making regarding the vaccine. WHO had a score similar to the one of funding agencies. On contrast, WHO got the highest score as the actors with interest at stake. WHO is the organization that most look at as a leader in GHG. Its main domain is providing policies and guideline. WHO has very limited power over other actors in the GHG field, thus in the decisions regarding the vaccines it scored lower than the governments and the vaccine manufacturers. WHO’s low level of authority over other actors contributes to the inadequate GHG performance.

Within the GHG system, the panel has scored two other important areas that affects the performance, the regulatory framework and the underlying values and priorities. As for the regulatory framework, the panel had the highest consensus on two aspects: the need for better enforcement of the IHRs, and the need for updating the IHRs and have more laws and regulations to regulate GHG actors, their contributions and interaction. IHRs are laws to control infectious disease, they are concerned with global surveillance and reporting system and set national minimum mandatory controls to prevent disease. In Covid − 19, there were many violations of the IHRs highlighting their weaknesses [30]. As for values and priorities, the panel agreed that market-oriented health norms are the norms affecting GHG decisions and actions concerning Covid-19 vaccines. Covid-19 is a global threat that affect all nations, to survive such a threat, health ought to be considered a global public good [31]. Perceiving health as a global public good entails that health resides beyond the authority of any one country and that people cannot be excluded from consuming such goods, nor does one person’s consumption of such goods should preclude consumption by another [32].

Inadequate performance and the underlying issues raise the question of what might be altered to improve future GHG performance. Clarifying who is the steward in GHG is one of the agreed upon future modifications to GHG. A steward is the actor responsible for setting priorities, building consensus, setting rules, and evaluating members and promote solidarity. WHO is considered the steward in GHG [33]. According to the analysis, it appears that this demand for clear stewardship is related to what took place during the pandemic such as the inadequacy in managing Covid-19 vaccine procurement, their inequitable distribution, use of political power to influence decisions concerning Covid-19 vaccines, and having a legal instrument to assure accountability leading to the call for better IHRs enforcement.

Accountability is another area that needs modification in GHG. Following the ineffective response to Covid-19, there is a call for “collective responsibility and mutual accountability” [34]. The findings of this study suggest that enhanced future accountability is related to transforming the GHG system to create a collective response to the pandemic, improving the inadequate management of vaccine avoiding unequal opportunities of procurement, accompanied by reviewing the absence of legal instrument to ensure equity.

Another area that needs improvement in GHG is authority. There were no clear calls for centralized authority in GHG raised in the pandemic. Some called for centralized Covid-19 data collection where data is to be merged under a centralized authority [35]. However, there was consensus from the panel that authority is better centered in GHG; this notion of centralization was found related to the need for more laws to regulate the actors and the fact that WHO can influence Covid-19 vaccines decisions and the use of cultural capital as form of power in GHG. This indicate that the type of centralized authority needed is to be expressed through laws and cultural capital managed by a neutral actor (e.g., WHO).

Equitable representation of actors had consensus as the most import GHG structural change to take place in the future. The worse the capacity of GHG to produce a collective response to Covid-19: inclusive decisions, facilitated solidarity, taking into consideration certain countries’ vulnerability, promoting health as a global public good and manage Covid-19 in a manner to achieve equity, the higher the demand for better representation of the global south in GHG. Also, the need for more equitable representation stems from the fact that GHG structure is loose with no legal instrument to ensure equity and accountability allowing vaccine manufacturers to influence decisions regarding Covid-19 vaccines knowing that pharmaceutical companies main interest is financial gain.

Similarly, to the previously mentioned modification – better representation of the global south in GHG – the need for a better legal framework is correlated negatively with Covid-19 vaccine production and equitable distribution and with the fact that health is not treated as a global public good. The worse the vaccines are managed and the less the reliance on global public good concept in dealing with health matters the more the demand for a better legal framework. On the other hand, the need for a better legal framework is positively correlated with the fact that GHG structure is loose with outdated IHRs and where vaccine manufacturers are decision makers regarding the Covid-19 vaccines and political power is used to influence these decisions.

### Study limitations

Despite being a reliable method to evaluate degrees of consensus on particular issues, the Delphi method has its limitations. Among the primary issues is gathering a panel of experts that is truly representative. For the current study, the panel experts were chosen carefully to have a certain level of expertise in the field and to represent the whole range of actors in GHG. Another issue is the choice of the Delphi survey statements. Identification of areas of concern representing the main problem and the construction of statements for these areas is a challenge in a Delphi survey. In the present study, statements were based on the results of a peer reviewed published systematic scoping review [17] which aimed at identifying areas of concern in GHG, equity and Covid-19. As for the statements, they were pilot tested on three experts for language, structure and comprehensibility.

## Conclusion

The GHG general performance as well as its performance in managing Covid-19 vaccine from its production to its distribution and procurement was not adequate. GHG performance limited the achievement of Covid-19 vaccines global equity. GHG performance is a product of the existing GHG system, its actors and legal framework. It is a collective result of individual GHG actors’ performance. The most influential actors in decision making regarding Covid-19 vaccine are the vaccine manufacturers and governments. While the most invoked power to influence decision are economic and political powers. Covid-19 decisions underlying value, although had human right to health at base, overlooked the concept of health as a global public good and were skewed towards market-oriented values. GHG malperformance along for its underlying factors calls for four main changes in GHG structure: assigning a clear steward for GHG, enhanced accountability, centralized authority, more equitable representation of actors, and better legal framework.

## Data Availability

The datasets used and/or analyzed during the current study are available from the corresponding author on reasonable request.
